# Multiplex Evaluation of Biointerface-Targeting Abilities and Affinity of Synthetized Nanoparticles—A Step Towards Improved Nanoplatforms for Biomedical Applications

**DOI:** 10.3390/molecules29225270

**Published:** 2024-11-07

**Authors:** Mélanie Romain, Céline Elie-Caille, Dorra Ben Elkadhi, Olivier Heintz, Michaële Herbst, Lionel Maurizi, Wilfrid Boireau, Nadine Millot

**Affiliations:** 1 Laboratoire Interdisciplinaire Carnot de Bourgogne, UMR 6303 CNRS—Université de Bourgogne, 21078 Dijon, France; melanie.romain@u-bourgogne.fr (M.R.); dorra.ben-elkadhi@u-bourgogne.fr (D.B.E.); olivier.heintz@u-bourgogne.fr (O.H.); michaele.herbst@u-bourgogne.fr (M.H.); lionel.maurizi@u-bourgogne.fr (L.M.); 2 Institut FEMTO-ST, UMR 6174 CNRS—Université de Franche-Comté, 25030 Besançon, France; celine.elie@femto-st.fr

**Keywords:** synthesis, gold nanoparticles, SPIONs, functionalization, thioglycolic acid, phosphonoacetic acid, mercaptohexanoic acid, PEG, theranostics, biofunctionalization, AFM

## Abstract

To obtain versatile nanoplatforms comparable for various bio-applications, synthesis and functionalization of two inorganic nanoparticles (NPs), i.e., gold (AuNPs) and iron oxide (SPIONs), are described for different NP diameters. Chosen ligands have adapted chemical function to graft to the surfaces of the NPs (thiols and phosphonates, respectively) and the identical frequently used external carboxyl group for comparison of the NPs’ material effect on their final behavior. To further evaluate molecular length effect, AuNPs are functionalized by different ligands. Numerous characterizations highlight the colloidal stability when grafting organic molecules on NPs. The potentiality of the functionalized NPs to react efficiently with a protein monolayer is finally evaluated by grafting them on a protein covered chip, characterized by atomic force microscopy. Comparison of the NPs’ surface densities and measured heights enable observation of different NPs’ reactivity and infer the influence of the inorganic core material, as well as the NPs’ size and ligand length. AuNPs have higher affinities to biomolecules, especially when covered by shorter ligands. NP ligands should be chosen not only based on their length but also on their chemical chain, which affects proteic layer interactions. This original multiplex comparison method using AFM is of great interest to screen the effects of used NP materials and functionalization when developing theranostic nanoplatforms.

## 1. Introduction

Many types of nanoparticles have been developed for biomedical applications. Among them, superparamagnetic iron oxide nanoparticles (SPIONs) and gold nanoparticles (AuNPs) are generating a lot of interest in the scientific community, and have already led to numerous publications (more than 150,000 and 28,000 of results on Web of Science, respectively since 2000) and several clinical studies (56 and 31, respectively, according to www.clinicaltrials.gov, accessed on 15 July 2024) [1,2,3,4,5]. Both have been developed for theranostics applications [6] or for chemical and biological sensing [7]. It is both their innocuity and their physical properties that are of interest for this type of application. SPIONs are developed for their magnetic properties (T2 contrast agents in MRI, magnetothermy, photothermy, magnetic targeting, cell sorting, etc.). AuNPs are largely developed for their plasmonic properties and their high atomic number (contrast agent in computed tomography, photothermy, radiosensitizer, biosensor, etc.). For some applications, both may be interesting; for instance, to treat cancer with hyperthermia [8,9]. They also can be associated in a single nanohybrid to combine their properties and develop a magneto-plasmonic object [10,11,12].

Synthetic raw inorganic nanoparticles require surface modification to be suitable for bio-applications. Indeed, to ensure their colloidal stability, it is necessary to perform a prefunctionalization step with organic molecules such as alkoxysilanes, phosphonates, citrates, cathecol derivatives, etc. [13,14]. Phosphonic acids and their derivatives are very interesting due to their strong affinity for hydroxylated surfaces [15]. The chemisorption of phosphonate agents on inorganic NPs is influenced by parameters such as temperature, pH, concentration, solvent, and type of oxide [16]. The phosphonates are also particularly interesting for SPIONs functionalization, in comparison with the traditionally used alkoxysilanes, because they form stable monolayers. They are less likely to detach from the surface of the oxide by self-condensation reactions, which can break the formed bonds. Gold NPs can be stabilized by citrates, such as in their synthesis by the Turkevich–Frens method, where chloroauric acid is reduced by sodium citrate that also acts as a stabilizer [17,18,19]. Since citrates are grafted via electrostatic interactions, ligand exchange is then facilitated. Thiol derivatives are very often used due to their high affinity with gold. However, maintaining electrostatic repulsions between NPs during this ligand exchange step is still challenging and parameters of this functionalization step should be carefully optimized [20].

The ligands mentioned before have terminal groups allowing the NPs to be stabilized in suspension, depending on the pH, due to the presence of charged functions on their surface (COO^−^, NH^3+^, Cl^−^, etc.). To compare their potential for different applications (pathology targeting, contrast power, heating power, etc.), it is interesting to compare and optimize several of the parameters previously discussed, such as the length of the alkyl chain, and the size and the nature of the inorganic core. In this study, the chemical nature of the NPs surface was deliberately fixed. Indeed, the will to study the surface charge of a nanoplatform does not always make much sense since these NPs are immediately covered with biomolecules, via the so-called protein corona, as soon as they are injected in a biological medium (in vitro or in vivo [21]). The behavior of these different nanohybrids in a biological system can then be compared and analyzed, in vivo, in vitro, or in silico [22].

In this paper, description of the steps to go through those model nanomaterials with the same “external” characteristics are described. Iron oxide (SPIONs) and gold nanoparticles (AuNPs) are synthetized, functionalized, and fully characterized to be able to finally compare material and ligand effects on their capability to interact with a biointerface. SPIONs of different oxidation states (magnetite and maghemite) and sizes (16 and 30 nm) were functionalized with phosphonate molecules. AuNPs were obtained with sizes in the same size range of SPIONs and their surfaces modified with thiolated molecules of different lengths (with 1 C or 5 C containing aliphatic chain and around 68 units containing polyethylene glycol (PEG) chain) terminated by a carboxylic group. These NPs with various sizes and surface chemistries reacted, in a multiplex format, on a biochip surface functionalized with albumin, the most abundant plasmatic protein [23], and were fully characterized in terms of grafting surface density and metrology by atomic force microscopy (AFM). The density of NPs after grafting on the surface, as well as the height of NPs, enable information to be extracted related to the efficiency of the NP functionalization procedure and the behavior of the NPs in terms of reactivity to a protein biointerface. Thus, the influence of the ligand molecular length, of the NP size, and of the inorganic core on NPs versus biomolecule interactions is finally discussed.

## 2. Results

### 2.1. Nanoparticles Synthesis

**Figure 1:**
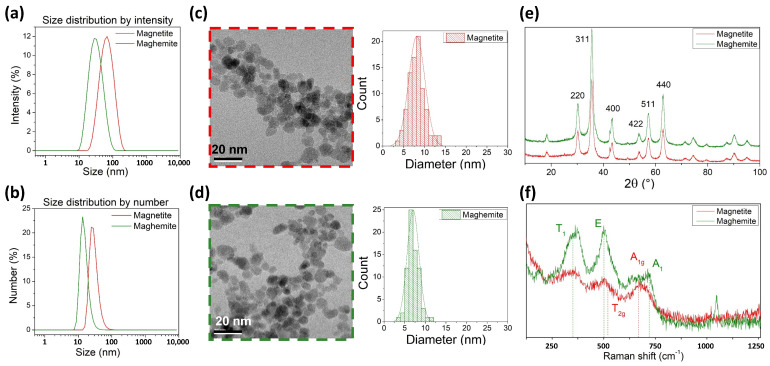
Size distribution of the SPIONs suspensions obtained by DLS (**a**) by intensity and (**b**) by number. TEM picture and size distribution of (**c**) magnetite NPs and (**d**) maghemite NPs. (**e**) XRD pattern and (**f**) Raman spectra of synthesized SPIONs.

Characterizations of the synthetized SPIONs are presented in Figure 1.

Monodispersed NP suspensions were obtained as shown in Figure 1a,b. Indexation of the different planes on the X-ray diffraction (XRD) pattern in Figure 1e corresponds to the cubic inverse spinel structure of the magnetite (ICDD: 04-021-3968, space group Fd3m). Lattice parameters were calculated and listed in Table 1, and correspond to those reported in the literature for such materials [24,25,26]. The lattice parameter of Fe_3_O_4_ NPs, different from the theoretical value of 8.396 Å, proves that NPs are slightly oxidized. The value of the deviation from oxygen stoichiometry δ, for NPs with a formula of Fe_3(1−δ)_O_4_, determined from the lattice parameters confirms this (δ = 0.053) [27]. Raman spectrum profiles in Figure 1f enable differentiation between the two different oxidation forms of iron oxide obtained, with characteristic A_1g_ and T_2g_ bands at 668 cm^−1^ and 515 cm^−1^ for magnetite and A_1_, E, and T_1_ bands at 720 cm^−1^, 500 cm^−1^, and 350 cm^−1^ for maghemite [28,29]. A shoulder at 720 cm^−1^ appears on the Raman spectrum of the magnetite powder; this contribution arises from a thin layer of maghemite at the surface of the magnetite NPs [26]. The differentiation can also be achieved by comparing the amount of iron relative to that of oxygen in both oxidation states by X-ray photoelectron spectroscopy (XPS) analysis (see Figure S1). Quantifications of total Fe (from 2p level) and O^2−^ contribution of the O 1s peak enable reaching Fe/O ratios of 0.82 and 0.71 for magnetite and maghemite, respectively. Those theoretical values for Fe_3_O_4_ and γ-Fe_2_O_3_ are 0.75 and 0.67, which is the same trend as the calculated one. Nevertheless, no difference is observed in the high-resolution spectra of Fe 2p, particularly in the binding energy difference between Fe 2p_3/2_ and its satellite peaks, which is approximately 8.4 eV for all the samples (Figure S1). This value is related to the oxidation state of the cation, depending on whether Fe^2+^ (6 eV) or Fe^3+^ (8 eV) is present in the samples [30]. Consequently, XPS cannot be used to determine the deviation from oxygen stoichiometry in our samples that have been exposed to air atmosphere, prior to XPS study. Concerning morphology aspect, both types of iron oxide nanoparticles are composed of single crystallites of almost 10 nm visible by transmission electron microscopy (TEM) in Figure 1c,d, which aggregate to form larger particles owing to the size obtained in dynamic light scattering (DLS). Size values measured with TEM and DLS or calculated from XRD are summarized in Table 1.

Sizes of the nanoparticles in suspension obtained from DLS are of around 30 nm for magnetite NPs and 16 nm for maghemite nanoparticles. The crystallite size calculated from the XRD pattern is slightly different from the observed measurement achieved on single crystallites observed with TEM, yet very close and in the same order of magnitude. However, DLS calculates the size of objects in suspension through optical phenomenon, and larger sizes are obtained. This is due to the fact that SPIONs prepared by Massart’s method are not isolated crystallites but small aggregates of several smaller crystallites. Thus, TEM and XRD provide precision on the crystallite sizes, forming particles whose sizes are 16 and 30 nm according to DLS.

**Scheme 1:**
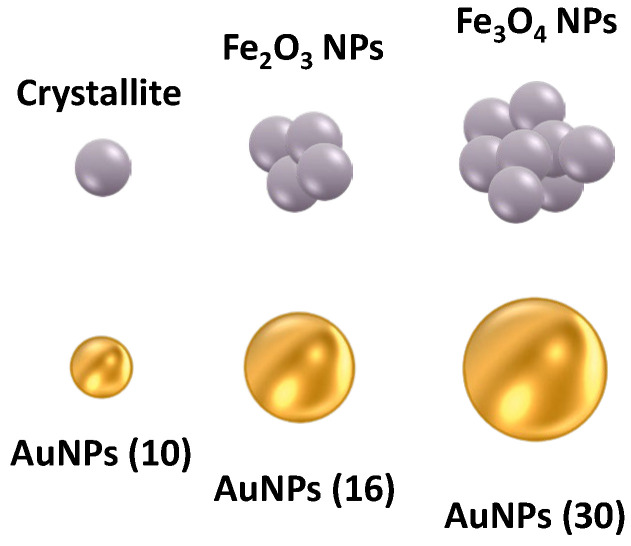
Schematic representation of the synthetized NPs with different sizes for AuNPs to mimic the obtained sizes of the SPIONs species.

In order to compare SPIONs with nanoparticles of another type of inorganic core, it is important to limit as much as possible the number of factors that can influence the final objects properties. Therefore, gold nanoparticles of different sizes (i.e., 10, 16, and 30 nm), corresponding to the size of crystallites and aggregates determined by DLS for maghemite and magnetite nanoparticles, respectively, were synthesized as represented in Scheme 1.

**Figure 2:**
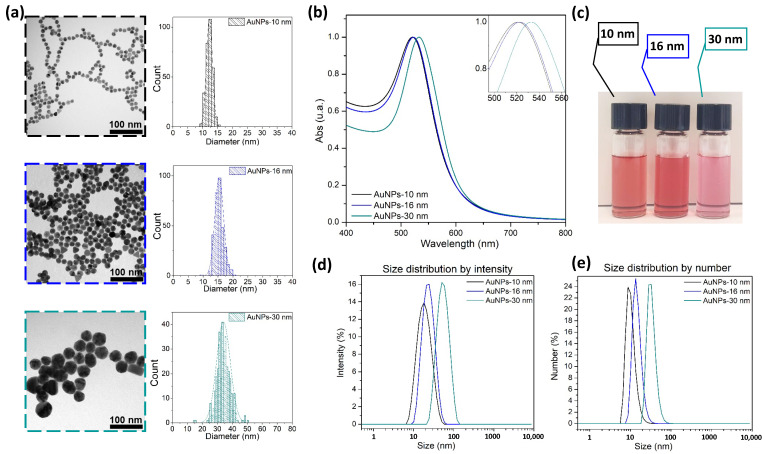
(**a**) TEM picture and size distribution of different sized AuNPs suspensions. (**b**) Normalized UV-visible spectra of different suspensions. Inset shows a zoom-in of maximum LSPR peak relative to different NP sizes. (**c**) Pictures of the AuNP suspensions and corresponding size distributions obtained by DLS (**d**) by intensity and (**e**) by number.

The different sized AuNPs were obtained by varying the ratio of citrate amount as the reducer over the gold amount to impact LaMer’s model of nucleation and growth [31,32]. Characterizations of the different sized suspensions are presented in Figure 2.

The obtained gold nanoparticles are faceted spheres as shown in Figure 2a, and the measured sizes listed in Table 2 show that the desired sizes are obtained with the different citrate: Au ratios.

With value uncertainties, the desired AuNP sizes were obtained in good correspondence between TEM and DLS values, and correspond to the sizes of the SPIONs in suspension that were targeted. Figure 2b,c show the evolution of the LSPR band with the NP size and the resulting color evolution. The resulting sizes, calculated from UV-visible spectroscopy according to Haiss et al. [33], are consistent with the obtained sizes in DLS or TEM, except for 30 nm NPs for which twice the size was obtained. It was observed that the calculation model does not enable always reaching relevant values due to some dependence on measurement volume. Finally, Figure 2d,e show a good monodispersity of each suspension, attesting the relevance and robustness of this protocol to tune the nanoparticle sizes when the aim is to target precise ones to be comparable to other materials.

### 2.2. Nanoparticles Functionalization

**Scheme 2:**
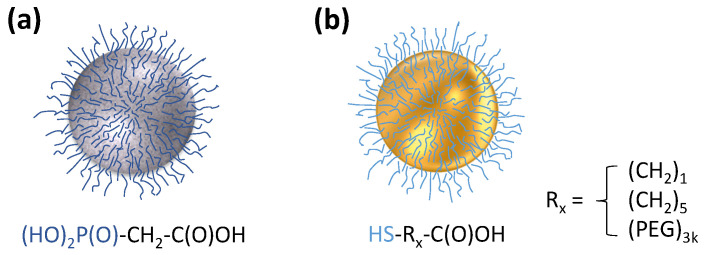
Schematic representation of the functionalized NPs of (**a**) iron oxide and (**b**) gold with ligand description.

Stabilization of the nanoparticles was then achieved with the use of a phosphonated ligand to attach to the iron oxide surface and a thiolated one for the gold surface, as shown in Scheme 2. The rest of the chain was identical to make the nanoplatforms comparable for further use, but different ligand lengths were also used on AuNPs to compare their final influence on the grafting.

**Figure 3:**
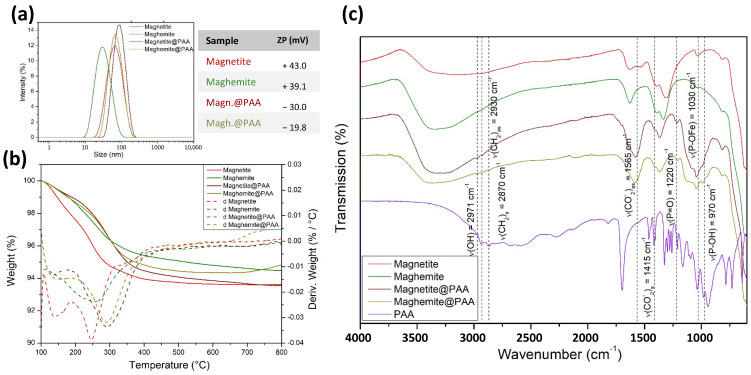
(**a**) Size distribution in intensity of the SPIONs suspensions (naked or modified by PAA) obtained by DLS and associated zeta potential at pH = 4.7. (**b**) TGA of the dried suspensions and (**c**) FTIR spectroscopy of the dried suspensions and initial precursor.

The functionalization process occurs by ligand exchange in both cases. Characterizations of the functionalized SPIONs with phosphonoacetic acid (PAA) are presented in Figure 3.

The resulting suspensions after functionalization are still monodisperse according to the DLS profile in Figure 3a, and a change in surface charge tends to indicate the covering of the positive SPION surfaces at this pH by the negatively charged external –COO^−^ groups. The mean hydrodynamic diameter goes from 30 ± 10 nm for magnetite to 52 ± 16 nm for magnetite@PAA, and from 16 ± 5 nm for maghemite and 38 ± 11 nm for maghemite@PAA, indicating an important size increase. Thermal decompositions in Figure 3b are delayed for the functionalized nanoparticles due to the presence of the additional organic layer to decompose. Nevertheless, since the final weight loss is approximately the same, it seems that the quantity of PAA grafted is, in mass, approximately the same than that of nitrates removed. However, the shift in the derivative peak of thermal decomposition from SPION curves to functionalized ones seems to indicate that a different species is present on the samples. FTIR spectroscopy attests the presence of the PAA molecules around the grafted NPs in Figure 3c where different bands can be assigned to the phosphonate group, the carbon chain, or the external carboxylic group. Indeed, stretching of P=O can be found at 1220 cm^−1^, when the stretching of the P-OH of the initial PAA at 970 cm^−1^ is only partially present in the grafted SPIONs and shifted to the stretching band of P-O-Fe at 1030 cm^−1^ depending on the conformation of grafting [14]. Symmetric and antisymmetric stretching of the CH_2_ in the PAA chain are visible at 2870 cm^−1^ and 2930 cm^−1^, respectively, and symmetric and antisymmetric bands of the carboxylic group stretching are found at 1415 cm^−1^ and 1565 cm^−1^, respectively.

**Figure 4:**
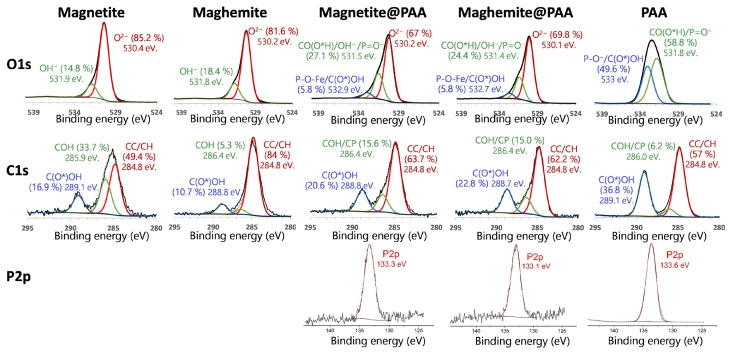
Fitted curves of the O 1s, C 1s, and P 2p peaks of the XPS spectra of the SPIONs, functionalized SPIONs, and initial functionalization molecule.

XPS analysis, in Table 3 and Figure 4, enabled additional evidences to be obtained on the efficacy of the functionalization step, with the apparition of phosphorous in the elemental composition of the functionalized SPIONs.

The interesting point is the changing contributions in the different elemental peaks, especially the apparition of the contribution of P-O-Fe and COOH bounds in the O 1s spectra, proving the PAA grafting on the surface of SPIONs (Figure 4). We can notice that the areas of the contributions P-O^−^/C(O×)OH and CO(O×H)/P=O^−^, which are in similar proportions in the PAA spectrum (41% and 59%, respectively), change when the molecule is grafted at the surface of the NPs. The P-O-Fe/C(O×)OH contribution decreases, to the benefit of that of CO(O×H)/OH^−^/P=O^−^, certainly due to the presence of remaining OH^−^ on the surface of magnetite and maghemite NPs [34,35]. The C 1 s peak consists mainly of environmental pollutions for the naked-SPIONs samples, but contributions change for the functionalized samples, with a strong increase of a contribution at 286.4 eV related to C-OH and C-P bounds [35]. As previously explained for bare SPIONs, no difference is observed in the high-resolution spectra of Fe 2p, in particular in the binding energy difference between the Fe 2p_3/2_ and its satellite peaks which is approximately 8.4 eV for all the SPIONs@PAA samples (Figure S1).

Finally, the ratio of concentration of Fe and P obtained by ICP measurements in Table S1 enabled calculation of the amount of PAA grafted molecules, which equals around 7 molecules/nm^2^ for the magnetite and 4 molecules/nm^2^ for maghemite, which is coherent with what can be found in the literature [36]. All those techniques allow for obtaining complete information on the functionalized NPs and to confirm the efficiency of the grafting protocol on both type of iron oxides.

**Figure 5:**
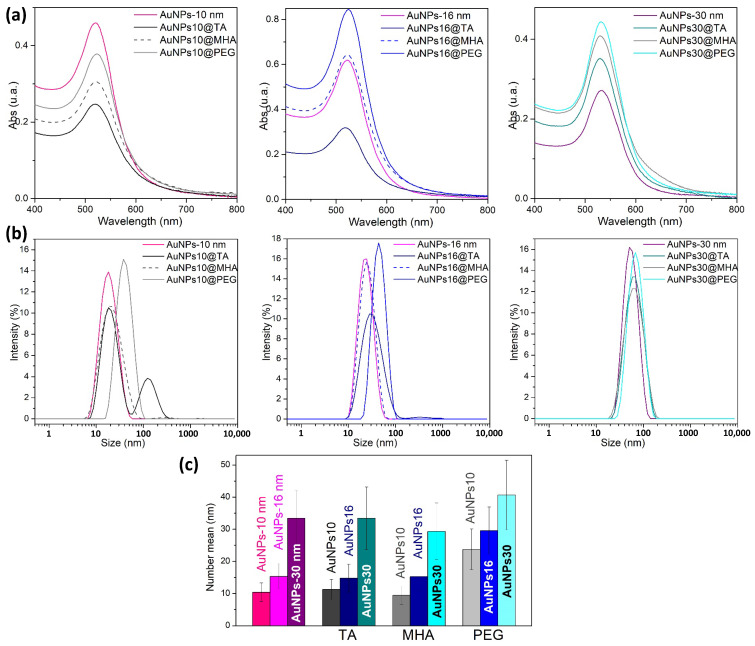
(**a**) UV-visible spectra of AuNPs suspensions of the different sizes before and after functionalization with the different ligands. (**b**) DLS size distribution by intensity and (**c**) resulting number mean hydrodynamic diameter of the AuNPs.

Characterizations of the functionalized AuNPs with the different ligands, thioglycolic acid (TA), 6-mercaptohexanoic acid (MHA), and HS-PEG(3k)-COOH (PEG) are presented in Figure 5.

First, LSPR profiles of all the suspensions attests of the maintained plasmonic properties of the suspensions. The size distribution presented in Figure 5b shows that monodispersity of the suspensions is maintained after ligand grafting with a slight size increase, except for the 10 nm AuNPs functionalized with TA where a second population appears above 100 nm. This second peak probably witnesses a partial aggregation of the sample resulting from the washing step. However, when looking at LSPR profiles in Figure 5a, no significant widening of the peak is observed. This indicates that this partial aggregation does not apply to the whole sample and does not affect final plasmonic properties. The same partial aggregation is slightly observed for 16 nm AuNPs functionalized with TA, but for no other sized AuNPs or when using MHA or PEG as a ligand. The longer chain probably helps in stabilizing the particles, especially for PEG, whose ethylene glycol chain is more hydrophilic [37]. Final hydrodynamic diameters in number distribution are reported in Figure 5c. They show an important size increase for PEGylated AuNPs compared to citrate capped ones. No significant size changes are observed in the case of TA- or MHA-functionalized AuNPs, because the ligand size does not significantly differ from the citrate molecule size, which finally does not significantly affect the hydrodynamic size.

Samples were investigated using SERS to check ligand molecules presence.

**Figure 6:**
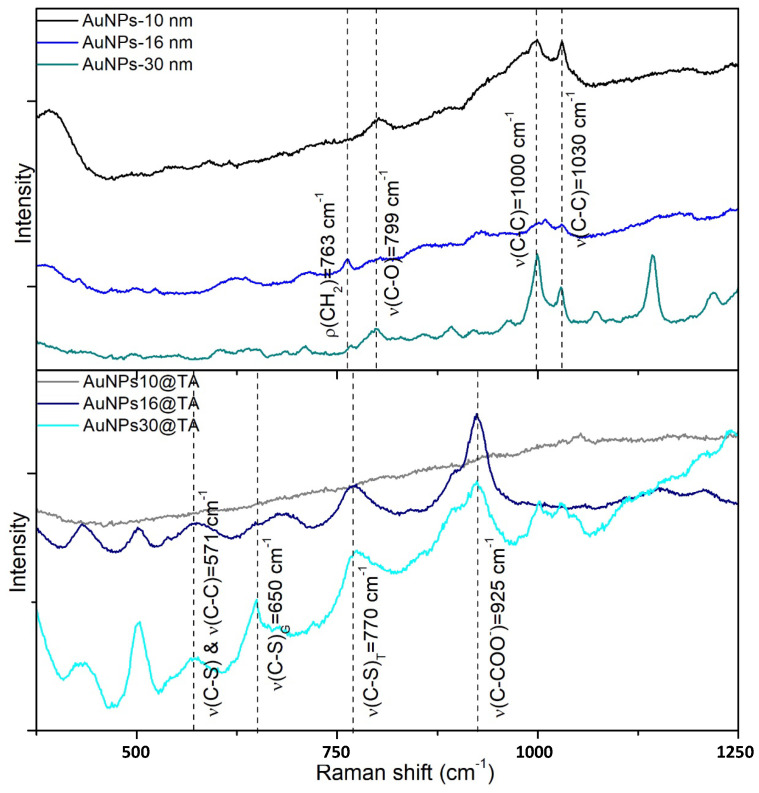
Raman spectra of different sized AuNPs suspensions before and after TA functionalization.

Resulting spectra in Figure 6 for TA-functionalized AuNPs show different vibrational modes compared to the as-prepared and functionalized AuNPs, but very similar results are obtained for the different sized suspensions. The different sized AuNPs stabilized with an excess of citrates molecules present particular bands at 1000 and 1030 cm^−1^ corresponding to C-C bonds stretching, but also at 799 cm^−1^ for the stretching of C-O bonding and at 763 cm^−1^ for the bending of CH_2_ that were previously described in the literature [38,39]. The functionalized AuNPs have more intense signals, except for the 10 nm particles that exhibit almost no peaks in that region. The two other different sized AuNPs present once again very similar profiles, with bands corresponding to ν(C-S) G and T conformers at 650 and 770 cm^−1^, respectively, that are not present on the initial sample stabilized with citrate. This confirms the introduction of the sulfur atoms from the thiolated ligand on the grafted AuNPs, as previously described in the literature [40,41]. Another band corresponding to a complex vibration between ν(C-C) and ν(C-S) is visible at 571 cm^−1^. Finally, an intense band at 925 cm^−1^ indicates the presence of C-COO^−^ bonding from the carboxylic external group, which confirms the presence of TA molecules for the 16 and 30 nm AuNPs. For the smallest sized AuNPs, the absence of those bands indicates a very poor grafting, probably responsible for the partial aggregation during the washing step observed with DLS results. The use of such a short molecule as TA with that procedure is thus not suitable for the grafting of small AuNPs of 10 nm. Other sized AuNPs present efficient functionalization and a maintained dispersion state that makes them suitable for further application. For MHA- and PEG-functionalized AuNPs, Raman spectroscopy in Figure S2 attests once again to the grafting of the thiolated molecules on the AuNPs. As for XPS, it is not appropriate due to the very low mass concentrations of our AuNPs suspensions. Sulfur is not detected (Figure S3). Nevertheless, a PEG-contribution at 286.7 eV appears in the C 1S contribution of the AuNPs30@PEG sample, in accordance with the study by D.J.H. Cant et al. (Figure S3) [42].

The two different types of SPIONs can thus be further compared with AuNPs with the same external chemistry, allowing for obtaining eventual information on the influence of the inorganic core and the NPs size.

### 2.3. Multiplex Evaluation of Grafting Ability Onto a Biointerface of the Functionalized NPs

**Figure 7:**
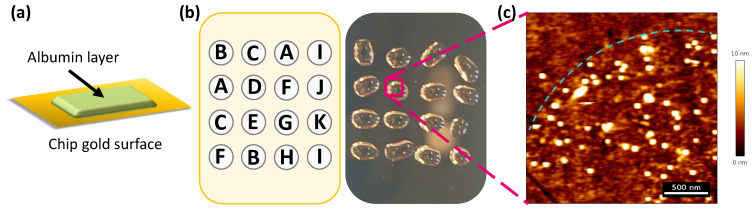
(**a**) Schematic representation of the protein layer covalently grafted onto a chemically functionalized surface of a gold chip. (**b**) Schematic representation of the deposited spots of A. Magnetite@PAA, B. Maghemite@PAA, C. Au10@TA, D. Au16@TA, E. Au30@TA, F. Au10@MHA, G. Au16@MHA, H. Au30@MHA, I. Au10@PEG, J. Au16@PEG, K. Au30@PEG, and picture of the spots obtained by droplet deposition of the different batches of activated functionalized NPs. (**c**) Zoom-in of the spotted surface observed with AFM, showing a spot border delimited by a dashed blue-line.

The two types of functionalized nanomaterials with different cores and diameters, but also molecular ligand length, were compared in terms of biomolecular grafting ability onto a biointerface of albumin. The building of the biointerface was monitored by SPRi: activated SAM by EDC/NHS was exposed to a solution of Rat Serum Albumin (RSA) under flow rate during several minutes of injection and the signal was monitored in real time. Thus, over all the surface exposed to the proteic solution, an average signal of 13.3 ± 0.4% of reflectivity variation was recorded. This corresponds to a surface molar coverage of 22 fmoles of covalently bound RSA per mm^2^, coherent with a highly packed and homogeneous protein monolayer. As it is crucial in the prospect of relative investigations of the reactivity of each type of NPs onto a biointerface, the SPRi monitoring of the biochip building ensures its good homogeneity and robust downstream analysis. To this aim of biomolecular grafting ability comparison, the different NPs, functionalized with carboxyl terminal functions, were activated and spotted in duplicate format onto this albumin protein layer in a multiplex format, as schematized in Figure 7.

**Figure 8:**
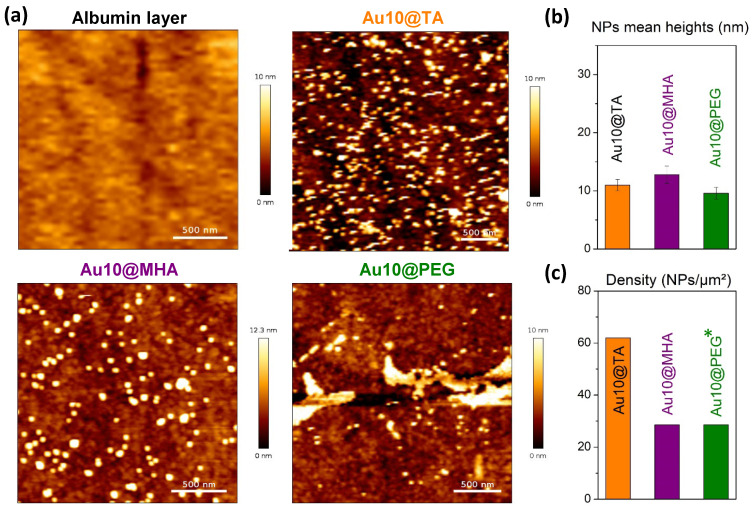
(**a**) AFM images of the albumin-covered chip, and of albumin-covered chip after grafting of 10 nm AuNPs with different ligands (TA, MHA, PEG). (**b**) Average heights measured on the visualized objects (number of measurements n = 60 for TA, n = 235 for MHA and n = 65 for PEG graftings) and (**c**) density of NPs observed on the surface. (*) Approximated counting due to packed arrangement of the NPs. Each image is representative of NP condition on their spots.

The spotted surface was then rinsed and dried prior to AFM analysis to observe, spot by spot, the quantity and sizes of the grafted objects. The analyzed surfaces spotted by 10 nm NPs are presented in Figure 8.

Except for the unspotted protein layer shown as the reference surface and for PEGylated AuNPs, the AFM images in Figure 8a show spherical objects homogeneously grafted on the surface, with heights between 9.6 and 12.8 nm according to measurements presented in Figure 8b. Thus, in all the cases, some activated functionalized 10 nm AuNPs were able to be grafted to the protein layer on the chip. Those results prove the ability of the functionalized 10 nm AuNPs to interact with a material (here, a biochip) covered with a protein layer, which opens the way to their use for bio-conjugation assays for further biomedical applications. This is also the case for Au10@TA NPs, despite their low pre-functionalization level identified thanks to Raman spectroscopy, so both grafting and adsorption may have occurred; this is highlighted by the upper level of grafting in comparison with other 10 nm NPs. The ligand influence on the NPs behavior can thus be compared. AuNPs functionalized with TA and MHA are randomly dispersed on the surface, while PEGylated AuNPs, except for few isolated particles, seem arranged according 2D dense packing, as specified in Figure S4. Since no aggregation was observed in the suspensions (Figure 5), and no evaporation procedure was applied on the spotted NPs, this auto-organization of the NPs happened during the incubation on the chip, driven by the coupling reaction. This may be due to decreased availability of activated carboxyl groups hidden in the long PEG chain, thus slowing down the coupling process to the biomolecules. In this case, high affinity between PEG chains onto different AuNPs may allow them to arrange in a bidimensional way (driven by the substrate) before or during grafting to the protein layer. When looking at the heights of the measured objects in Figure 8b, measured values are coherent with the initial NPs size. For PEGylated AuNPs, the long PEG chain would tend to increase the object diameter, yet a lower height is measured here (9.6 ± 1.0 nm). It appears clear that the interaction of such PEGylated AuNPs onto the proteic layer leads to different behaviors from those of the other NPs. The PEG chains affinity and the 2D NPs assembling could induce an interpenetration of the organic and inorganic structures, leading to an apparent height of around 10 nm. Finally, the density of NPs on the spotted protein surface is presented in Figure 8c. It must be noted that due to the packed arrangement of the PEGylated AuNPs, the counting to calculate density of individual nano-objects was delicate, so caution must remain for interpretation. It can be noted that, surprisingly, TA-functionalized AuNPs have higher density than MHA-functionalized ones. Indeed, the poor TA amount obtained after AuNPs 10 nm functionalization may have led to lower number of activated functions, and should have led to lower grafting amount on the biomimetic surface. However, this poor grafting leads to a weakly covered gold surface, probably favoring physisorption of the AuNPs onto the albumin layer, or even chemisorption with possible free cysteines of RSA, so for these reasons, this suspension should not be considered for its use in biomedical applications.

**Figure 9:**
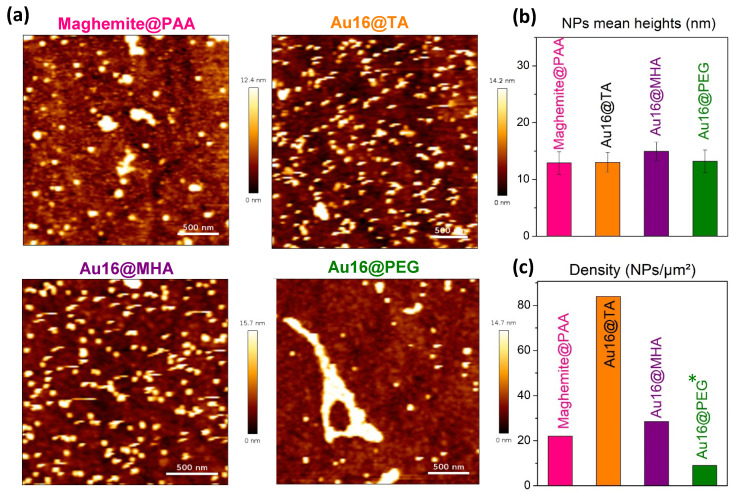
(**a**) AFM images of the albumin-covered chip after grafting of 16 nm NPs (maghemite and AuNPs) with different ligands (PAA, TA, MHA, or PEG). (**b**) Average heights measured on the visualized objects (number of measurements n = 85 for Maghemite@PAA, n = 80 for Au16@TA, n = 275 for MHA, and n = 110 for PEG graftings) and (**c**) density of NPs observed on the surface. (*) Approximated counting due to packed arrangement of the NPs. Each image is representative of NPs condition on their spots.

Then, 16 nm NPs functionalized with the different ligand were analyzed in the same way, as presented in Figure 9.

Once again, homogeneous repartition of objects is observed in Figure 9a, except for Au16@PEG species; however, all the NPs were able to graft on the albumin layer. Like previously, isolated NPs are observed for TA- and MHA-functionalized AuNPs, and PEGylated particles form a 2D film, confirming the specific behavior of AuNPs with this functionalization. A cooperative auto-assembling of the NPs on the surface must have occurred, and may start on a grafted NPs as nucleation point, acting like an anchoring platform, that initiates the growth of the highly organized 2D structure. For maghemite NPs, the observed objects are isolated spheres or small aggregates. This expected result is explained by the difference of dispersity state of the SPIONs suspensions versus the AuNPs ones, with SPIONs exhibiting wider size range in DLS intensity measurement than AuNPs; and also due to the fact that SPIONs are aggregates of single crystallites. The measured mean heights around 14 nm in Figure 9b are very close, and validate the relevance of the evaluation method for such objects. The NP densities on the surface are depicted in Figure 9c. First, with same ligand lengths for TA and PAA, it seems that AuNPs have higher grafting densities than SPIONs. This is assumed to be due to the higher affinity of gold to biomolecules, also arising from the possibility for gold atoms to interact with sulfur containing amino acids in RSA [43,44]. This higher affinity should not impact PEGylated AuNPs, highly passivated with such long ligand shell. This could explain the decreasing grafting density with the increasing ligand length chain, even though Au16@PEG density probably is underestimated due to the difficulty of differentiating single particles within the 2D NPs films.

**Figure 10:**
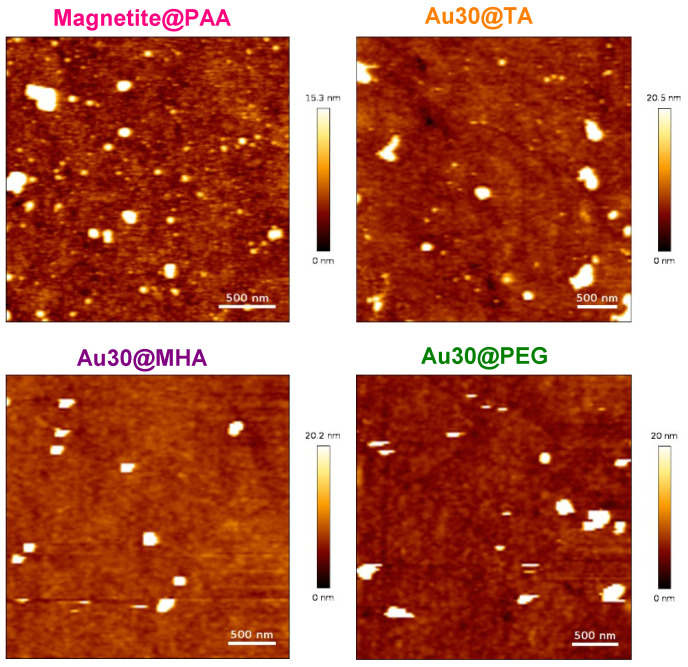
AFM images of the albumin-covered chip after grafting of 30 nm NPs (magnetite and AuNPs) with different ligands (PAA, TA, MHA or PEG).

Finally, the spots of the 30 nm NPs were analyzed by AFM, as presented in Figure 10.

The presence of all the types of NPs on the surface still proves their biomolecule grafting abilities. As the spotting was carried out with identical mass concentration, differences in the number of NPs were identified due to their size differences, which were more significant for 30 nm NPs. This explains the low number of objects observed on AFM images in Figure 10. Heights and densities of the observed NPs on the surface are available in Figure S5. Typical obtained average heights range from 10 to 15 nm for Magnetite@PAA and Au30@TA, and from 30 to 35 nm for Au30@MHA and Au30@PEG, with some heterogeneities in size within the observed objects. The 30 nm AuNP suspension was the one with the largest size dispersity in the measured population in TEM (Table 2), which would explain the larger differences observed. However, the low height value observed in the case of TA functionalization seems to result from the additional population of very small particles present on the surface. It can be seen that magnetite NPs present both very small sized objects and particle aggregates. As for maghemite NPs, the wider dispersity of NPs in suspension may explain that difference. Surprisingly, the amount of smaller particles seems more important in that case, which could eventually be attributed to a dispersion of some crystallites making up the SPIONs agglomerates, due to individual NPs/biomolecule interactions.

As previously shown, with an only one substrate bearing a proteic biointerface, very interesting information can thus be extracted from this original multiplex evaluation method to compare behaviors and biomolecular grafting abilities of NPs with different core materials, diameters, and ligand sizes. By controlling the quality of the proteic coverage with SPR experiments, we ensure that each type of functionalized NPs will face off a robust and homogeneous biointerface. Moreover, in situ characterization of the NP coatings, at the single NP scale, with AFM ensures a very accurate qualitative and quantitative investigations. As for all analytical approaches, the number of objects of interest has to be high enough to make the analysis robust, which can be achieved by defining adapted working concentration.

## 3. Materials and Methods

Materials. Tetrachloroauric acid trihydrate (HAuCl_4_.3H_2_O, 99.99%) was purchased from ThermoFischer Scientific (Waltham, MA, USA). Iron (III) chloride hexahydrate (FeCl_3_.6H_2_O, ≥98%), iron (II) chloride tetrahydrate (FeCl_2_.4H_2_O, ≥99%), iron (III) nitrate nonahydrate (Fe(NO_3_)_3_.9H_2_O, 97%), sodium hydroxide (NaOH, ≥97%), ammonium hydroxide solution (NH_4_OH, 28%), hydrochloric acid (HCl, 37%), and nitric acid (HNO_3_, 70%) were purchased from Sigma-Aldrich (St. Louis, MO, USA). Tri-sodium citrate trihydrate (C_6_H_5_Na_3_O_7_·2H_2_O, >99% FG), thioglycolic acid (TA, HSCH_2_COOH, >99%), 6-mercaptohexanoic acid (MHA, C_6_H_12_O_2_S, 90%) phosphonoacetic acid (PAA, (HO)_2_P(O)CH_2_COOH, 98%), N-(3-Dimethylaminopropyl)-N′-ethylcarbodiimide hydrochloride (EDC), Hydroxy-2,5-dioxopyrrolidine-3-sulfonate sodium salt (sulfo-NHS), Rat Serum Albumin (RSA), Phosphate Buffer Saline (PBS), and Octyl Glucopyranoside (OG) were purchased from Sigma Aldrich. HS-PEG(3k)-COOH was purchased from Iris Biotech. Ethanol absolute was purchased from VWR. All aqueous solutions were prepared with ultrapure water with a resistivity >18.2 MΩ.

Synthesis of the nanoparticles: SPIONs—Massart vs. AuNPs—Turkevich–Frens. AuNPs were synthetized by the Turkevich–Frens method. All glassware was pre-washed with aqua regia. A volume of 100 mL of a HAuCl_4_·3H_2_O solution was heated at 100 °C in a 250 mL two-necked round-bottom flask upon agitation under reflux. Then, 10 mL of a 0.030 M sodium citrate tribasic dihydrate solution was quickly added to the gold solution. The temperature was maintained for a defined duration, then stopped to let the mixture cool down to room temperature. The size of AuNPs was controlled by the mass ratio of gold salt to sodium citrate and reaction time. For the three sizes targeted, reaction times of 20, 60, and 100 min were set up for the three mass ratios of citrate:Au of 3.96:1, 8:1, and 19.5:1 respectively.

Two types of magnetic iron oxides were prepared: magnetite and maghemite. Both are superparamagnetic and are called SPIONs when distinction is not necessary. They were synthesized according to a protocol adapted from Massart [24,45]. Iron (III) chloride (FeCl_3_.6H_2_O) and iron (II) chloride (FeCl_2_.6H_2_O) were added in a Fe(III):Fe(II) molar ratio equal to 2:1 in water under stirring until complete powders dissolution, after which ammonia (NH_3_) was added to the precipitate magnetite. The whole was stirred for 2 min. The purification step of the oxides thus formed was performed by magnetic decantation. The supernatant was eliminated and the powder was resuspended in water. This washing was performed twice before final washing via dialysis in 10 mM nitric acid (HNO_3_). Isolation of nanoparticles was then carried out by centrifugation for 30 min at 10,000× *g*. The magnetite NPs were recovered in a supernatant that contained stable NPs. An oxidation step transformed the magnetite NPs into maghemite ones. In that case, a centrifugation step of 5 min at 5000× *g* was carried out right after magnetic decantation washings. After resuspension in 2 M nitric acid, a solution of iron (III) nitrate (Fe(NO_3_)_3_) was added to the suspension in a round-bottom flask, which was placed under reflux at 120 °C and stirred for 1 h 30 min. The flask was then cooled down before a mixture transfer to a beaker, which was washed by magnetic sedimentation, before following the previous steps of dialysis and isolation.

MA grafting. The grafting of mercapto acids (MAs) to the AuNP surfaces followed the ligand exchange mechanism. In a glass flask with stirrer, a 25.4 mM TA or MHA solution was injected into the as-prepared GNPs suspensions with a dilution factor of 1000, or a 5 mM PEG solution injected with a dilution factor of 200. The final mixture was stirred at 400 rpm at 25 °C in the dark for 1 h, before being washed by centrifugation (with time and speed depending of AuNPs size) and resuspended in ultrapure water.

PAA grafting. In a plastic flask with stirrer, a specific volume of SPIONs suspension was mixed with water to obtain a 1 mg/mL concentration. A certain volume of a prepared aqueous PAA solution was added to the SPIONs suspension to obtain a SPIONs:P molar ratio equal to 1:0.5. The pH was adjusted to 4 via the addition of a 0.1 M NaOH solution. The final mixture was placed under stirring at 400 rpm for one night, before being washed by dialysis (with a cut-off threshold of 12–14 kDa) in water. Sonication enabled dissociation of the agglomerates formed upon dialysis.

Nanoparticles grafting on a biointerface. A volume of 100 µL of the different suspensions of functionalized nanoparticles with a NP concentration of 23.7 µg/mL was activated by adding 2 µL of a fresh mixture EDC and sulfo-NHS at a concentration of 200 and 50 mM respectively, then mixed for 15 min. The different activated NPs were then spotted by injecting 300 nL through a mask on a chip previously covered with a homogeneous protein layer of albumin (see Methods section). The spotted chip was sonicated 30 min to favor the reaction between activated NPs and the free lysine of albumin. In this way, the chip finally presented a pattern of different NPs batches, grafted in arrays format (one array: 1 mm in diameter). Once the NPs were grafted, the chip was gently rinsed with buffer, then with water, and dried prior to AFM measurements. Two spots of each NP were prepared for analysis to ensure the repeatability of the experiments.

Methods. TGA (TA instrument, Discovery TGA, Newcastle, UK) was used to determine the amount of molecules on the SPIONs surface. All powders were analyzed with a temperature ramp of 10 °C∙min^−1^ from 100 to 800 °C under an airflow rate of 25 mL∙min^−1^.

Zeta potentials of NPs suspensions were measured with a Malvern Nano ZS instrument (Worcestershire, UK) supplied by DTS Nano V7.11 software. DLS measurements of NPs suspensions were performed at 25 °C on the same instrument using disposable cuvettes. Measurements were analyzed using a backscattering angle (173°) and repeated three times for each sample to calculate standard deviations. The used refractive index for SPIONs is 2.420 and the absorption equals 0.010. These values are respectively 0.200 and 3.320 for AuNPs.

UV-visible absorbance of AuNPs suspensions was measured using Shimadzu UV-2550 UV-visible spectrophotometer (Tokyo, Japan). Spectra were recorded from 400 to 800 nm for each suspension, poured in disposable cuvettes, after baseline recording with deionized water.

A PHI Quantes apparatus (ULVAC PHI, Kanagawa, Japan) from a monochromatic focalized Al Kα1 X-ray source (EKα1 (Al) = 1486.7 eV with a 200 μm diameter spot size and a photoelectrons emergence angle of 45° were used to record XPS measurements, with pass energies of 280 eV for general spectrum and 55 eV for high-resolution window. Sample preparation consisted of pressing SPIONs powders on an indium sheet and successive drops deposition and drying for AuNPs suspensions onto a silicon wafer. Data analysis and curve fittings were realized with CasaXPS processing, and MultiPak software (version 9.0.1) was employed for quantitative analysis [46]. Calibration of the spectrum were achieved on C 1s peaks (for CC/CH bond at 284.8 eV). A Shirley background was subtracted and Gauss (70%)–Lorentz (30%) shapes were applied for curve fitting. The charge effects were minimized by a neutralization process.

Nanoparticles morphology and size characterization were performed using a JEOL JEM-2100F (Tokyo, Japan), with an accelerating voltage of 200 kV, and fitted with an ultra-high pole-piece achieving a point-to-point resolution of 0.19 nm for SPIONs, and with a Hitachi HT7800 setup with an acceleration voltage of 100 kV for AuNPs. Samples were prepared by evaporating a diluted suspension of nanoparticles onto the carbon-coated copper grids. NP diameters were calculated as the mean of the measurement of 300 NPs using Image J software (version 1.8.0_172) [47].

X-Ray diffraction (XRD) patterns of bare SPIONs were obtained using a Bruker D8 Advance diffractometer. Cu Kα1,2 radiations (λα1 = 1.540598 Å and λα2 = 1.544426 Å) were applied. Scans were measured over a 2θ range of 10–100°. A step of 0.0307° and a scan speed of 52 s per angle unit were set. The data analysis was carried out with Topas^®^ software (version 6). The Le Bail method was used to obtain lattice parameters and mean crystallite size.

A Bruker Vertex 70v (Billerica, MA, USA) with OPUS version 3.1 software (Billerica, MA, USA) was used to record FTIR spectra of SPIONs lyophilized powders using ATR.

Raman spectroscopy measurements of lyophilized naked SPIONs powders and surface enhanced Raman spectroscopy (SERS) of dried deposited drops of AuNPs suspensions were performed using a Renishaw InVia microspectrometer. The 785 nm excitation wavelength of a diode laser and a ×50 microscope objective were used.

Determination of iron and phosphorous content in suspensions was performed by inductively coupled plasma optical emission spectroscopy (ICP-OES) analysis using an ICP-OES 5110 (Agilent Technologies, Santa Clara, CA, USA) coupled with ICP Expert 7.3. software. RF power was 1.5 kW, nebulization started at 0.7 L/min, and pump speed was set at 12 tr/min. SPIONs suspensions were dissolved in 2% chlorohydric acid and standard solution prepared were of 0, 30, 100, 170, 240, and 300 ppm of Fe and of 0, 0.5, 1.5, 2.5, 3.5, and 5 ppm for P in the same matrix. Concentration was determined with the mean of three replicates per sample.

For protein monolayer formation on the chip, we used SPR experiments that were carried out using a SPRiplex-II (Horiba Scientific, Irvine, CA, USA) instrument at a temperature of 25 °C. Home-made gold-biochips composed of a glass slide coated with a thin layer of chromium (2 nm Cr) and gold (48 nm Au) were made using Plassys DC magnetron sputtering at the “Mimento” technology center (Besançon, France). The chip surface was chemically functionalized by incubating the biochip in a mixture of 16-mercapto-1-hexadecanoic acid (16-MHA) and 11-Mercapto-1-undecanol (11-MUOH) (90/10 by mole) overnight under continuous agitation at RT, leading to a Self-Assembly Monolayer (SAM). The biochip was then washed with absolute ethanol and ultrapure water successively prior to drying. In order to immobilize the protein (Rat Serum Albumin—RSA), the functionalized surface of the biochip was incubated with a mixture of 200 mmol/L EDC and 50 mmol/L sulfo-NHS for 30 min, then washed with ultrapure water. The RSA immobilization on activated SAM was achieved as described and visualized previously [48,49]. SPR experiments were carried out in PBS buffer (running buffer) and grafting of RSA was monitored in acetate buffer (pH4.5) at 40 µg/mL following several cycles of 10 min to reach the saturation of the signal. The grafting levels of RSA were determined from the SPRi value converted into mass per surface unit (1% of reflectivity variation = 115 pg/mm^2^).

AFM measurements were realized with a JPK Nanowizard III, in contact mode, using triangular cantilevers of 200 μm length, 28 μm width, and a spring constant of 0.08 N/m. AFM characterization was realized in air on the dried albumin chips grafted by NPs. The methodology was to scan from 3–5 large areas (typically 10 × 10 µm^2^) to small areas (1 to 5 µm^2^), first to have a representative view and second to obtain the good resolution in metrology, of each NP batch. The mean diameter and height of the NPs were extracted from all the images of the NP batches, using Gwyddion software (version 2.65), choosing 8.5 nm as threshold.

## 4. Discussion and Conclusions

Nanoparticles of different inorganic cores were successfully synthetized with Massart’s protocol for SPIONs and Turkevich–Fens’ method for AuNPs. With variation of the sodium citrate over gold ratio, the AuNP sizes were modified to obtain comparable objects, according to DLS sizes, to the different SPIONs (maghemite or magnetite). All those different suspensions were then functionalized to ensure identical chemical reactivity using ligand with adapted chemical functions. A phosphonated ligand was thus used to attach to the iron oxide surface and thiolated ones were used for their affinity to the gold surface. The efficiency of functionalization was investigated with DLS, Zeta measurements, FTIR spectroscopy, TGA, and XPS for SPIONs, and the high ligand density on the surface was highlighted. For AuNPs, functionalization was characterized with DLS, UV-visible, and Raman spectroscopy, and showed efficient grafting and good dispersion state, except for the smallest sized AuNPs functionalized with the shortest molecule (Au10@TA).

The different materials and sizes of NPs were finally compared simultaneously in terms of their ability to graft onto a proteic monolayer after chemical activation. An original method was employed to compare it by assessing NP density after grafting on a homogeneous biointerface. Activated functionalized NPs were spotted on a gold chip bio-functionalized with albumin, and the obtained biochip was analyzed with AFM in a multiplex format. The final object densities enable favoring the use of gold over SPIONs for their higher affinity to biomolecules. AFM images also proved an important ligand influence on NPs behavior in the case of PEGylated AuNPs, which formed 2D films by an auto-assembling mechanism due to PEG chain interactions with the proteic layer. This behavior could be interesting for the community working on nanocrystals assembling, since it was shown in some cases that ligands have an ability to influence 3D conformation [50,51,52,53]. Comparison of the measured objects heights and counting enables evaluation of the reactivity of the ligands to the biointerface.

The scope of this study is to offer protocols for the preparation of versatile nanoplatforms, and to propose a method for their reactivity testing and comparison of materials. This comes into play in the hybrid NPs preparation phase, before the possibility of applications of the final nanoplatforms. The nano-QSAR is also a very promising technique [22] but should be used after the development of the final nanoplatforms that this article is supposed to help with. This work enables testing and selecting efficient protocols for the synthesis and functionalization of the most commonly used NPs type, and presents a multiplexed and multimodal method of comparison of their performances. This ability of grafting with a biointerface and the interaction or repulsion triggered by the ligand used are crucial to know because these are necessary steps in most projects dealing with biomedical applications (targeting of a pathology, passing through a biological barrier, biosensing, etc.), especially in the field of theranostics where NPs often interact with biomolecules [54]. Such a nanoplatform supported by biophysical instrumentations offers tremendous perspectives in the screening of nanovectors and their targeting properties.

## Figures and Tables

**Table 1:** Lattice parameters and crystallite size calculated from XRD, crystallite size measured from TEM, and number mean size obtained from DLS of the synthetized SPIONs.

Type	a (Å)	XRD—Crist. Size (nm)	TEM—Crist. Size (nm)	Final DLS Size (nm)
Magnetite	8.371 ± 0.001	9.7 ± 0.1	8.2 ± 2.0	30 ± 10
Maghemite	8.353 ± 0.001	8.3 ± 0.1	7.0 ± 1.5	16 ± 5

**Table 2:** Characteristics of the synthetized AuNPs, with particle size measured from TEM, number mean size obtained from DLS, calculated size from UV-visible spectroscopy according to Haiss et al. method [33], and LSPR value.

Type	DLS Diameter (nm)	TEM Diameter (nm)	UV-Visible Size (nm)	LSPR (nm)
AuNPs (10)	10.4 ± 2.9	10.9 ± 1.0	12.1 ± 2.1	521.0
AuNPs (16)	15.3 ± 3.9	15.4 ± 1.5	16.2 ± 2.9	523.0
AuNPs (30)	33.5 ± 8.6	34.1 ± 4.6	62.4 ± 11.2	532.5

**Table 3:** Atomic concentrations obtained by XPS analysis from high-resolution photoelectron spectra for different elements in SPIONs, grafted SPIONs, and PAA.

Type	Fe 2p (%)	O 1s (%)	C 1s (%)	P 2p (%)
Magnetite	38.1	54.4	7.6	0
Maghemite	31.0	53.2	15.8	0
Magnetite@PAA	31.0	54.1	10.7	4.1
Maghemite@PAA	30.3	55.2	10.5	3.9
PAA	0	54.6	22.5	22.8

## Data Availability

The original contributions presented in the study are included in the article, further inquiries can be directed to the corresponding author/s.

## Supplementary Materials

The following supporting information can be downloaded at: https://www.mdpi.com/article/10.3390/molecules29225270/s1, Figure S1: XPS spectra of (a,b) the synthetized and functionalized SPIONs with PAA, (c) high-resolution spectra of Fe 2p, (d) table of peaks positions. Figure S2: Raman spectra of the AuNPs suspensions functionalized with MHA and PEG. Figure S3: XPS spectra of (a) AuNPs-30 and AuNPs30@PEG and (b) high-resolution spectra of C 1s and Au 4f of these samples. Table S1: Precision for the calculation of PAA densities on functionalized SPIONs from ICP-OES. Figure S4: AFM image of the albumin layer spotted by Au10@PEG NPs and zoom-in on a 2D pack of auto-assembled NPs, with height measurement. Figure S5: (a) Average heights measured on the visualized objects of AFM images of the spotted 30 nm NPs (Figure 10) and (b) density of NPs observed on the surface.
