# Supplementary material for: Multiplex Evaluation of Biointerface-Targeting Abilities and Affinity of Synthetized Nanoparticles—A Step Towards Improved Nanoplatforms for Biomedical Applications
Source: Molecules. 2024 Nov 7;29(22):5270. doi: 10.3390/molecules29225270 (PMC11596608; doi:10.3390/molecules29225270)
Supplement: Supplementary file 1 [file molecules-29-05270-s001.zip › molecules-3181132-supplementary.pdf]

molecules

Article

Multiplex evaluation of biointerface targeting abilities and affinity of synthetized nanoparticles – a step towards improved nanoplatforms for biomedical applications

Mélanie Romain [1], Céline Elie-Caille [2], Dorra Ben Elkadhi [1], Olivier Heintz [1], Michaële Herbst [1], Lionel Maurizi [1], Wilfrid Boireau [2,*], and Nadine Millot [1,*]

[1] Laboratoire Interdisciplinaire Carnot de Bourgogne, UMR 6303 CNRS/Université de Bourgogne, Dijon 21078, France
[2] Institut FEMTO-ST, UMR 6174 CNRS/Université de Franche-Comté, Besançon 25030, France
* Correspondence: NM: Nadine.Millot@u-bourgogne.fr; WB: Wilfrid.Boireau@femto-st.fr

Citation: To be added by editorial staff during production.

Academic Editor: Firstname Lastname

Received: date
Revised: date
Accepted: date
Published: date

Copyright: © 2024 by the authors. Submitted for possible open access publication under the terms and conditions of the Creative Commons Attribution (CC BY) license (https://creativecommons.org/licenses/by/4.0/).

Figure S1: XPS spectra of (a-b) the synthetized and functionalized SPIONs with PAA, (c) high-resolution spectra of Fe 2p, (d) table of peaks positions.

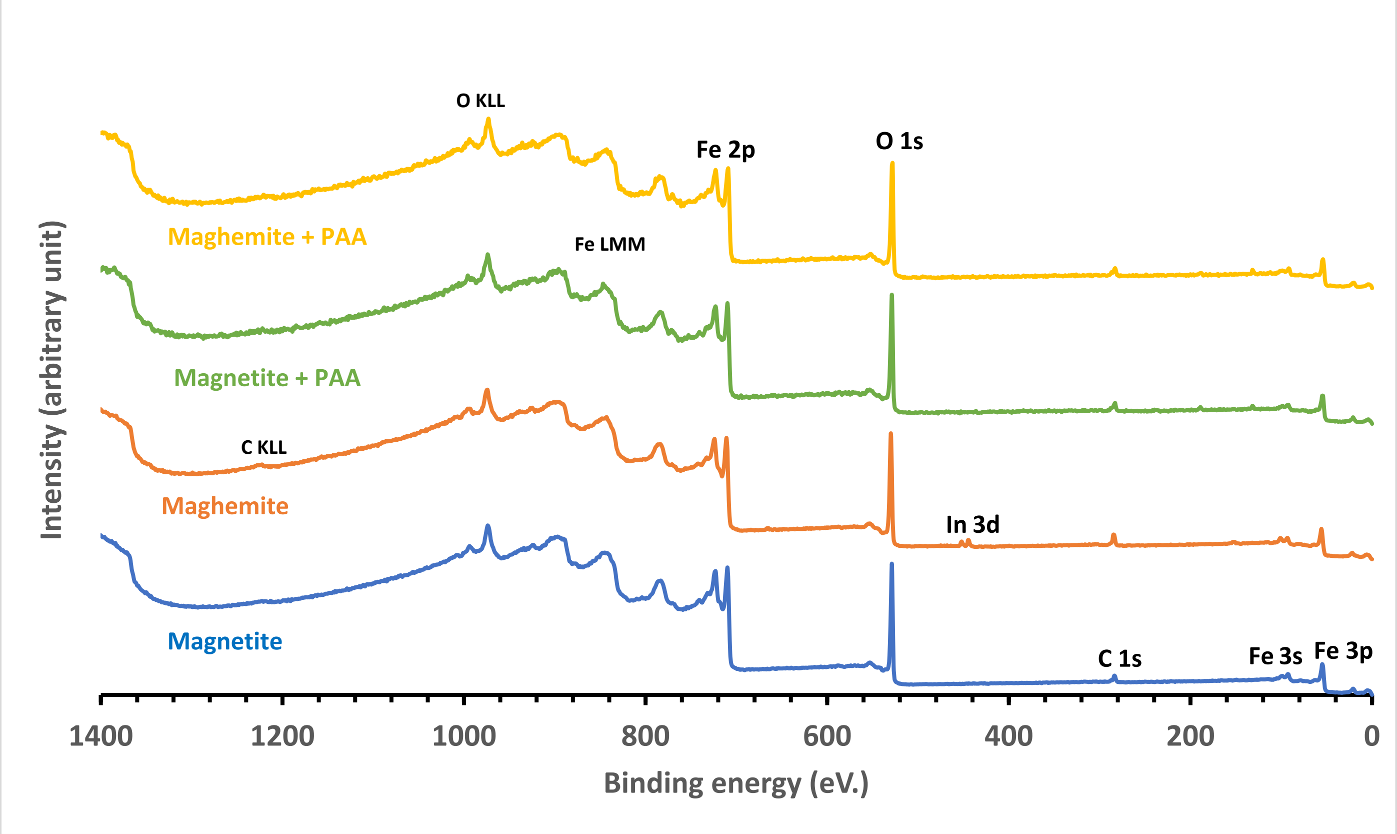

(**a**)

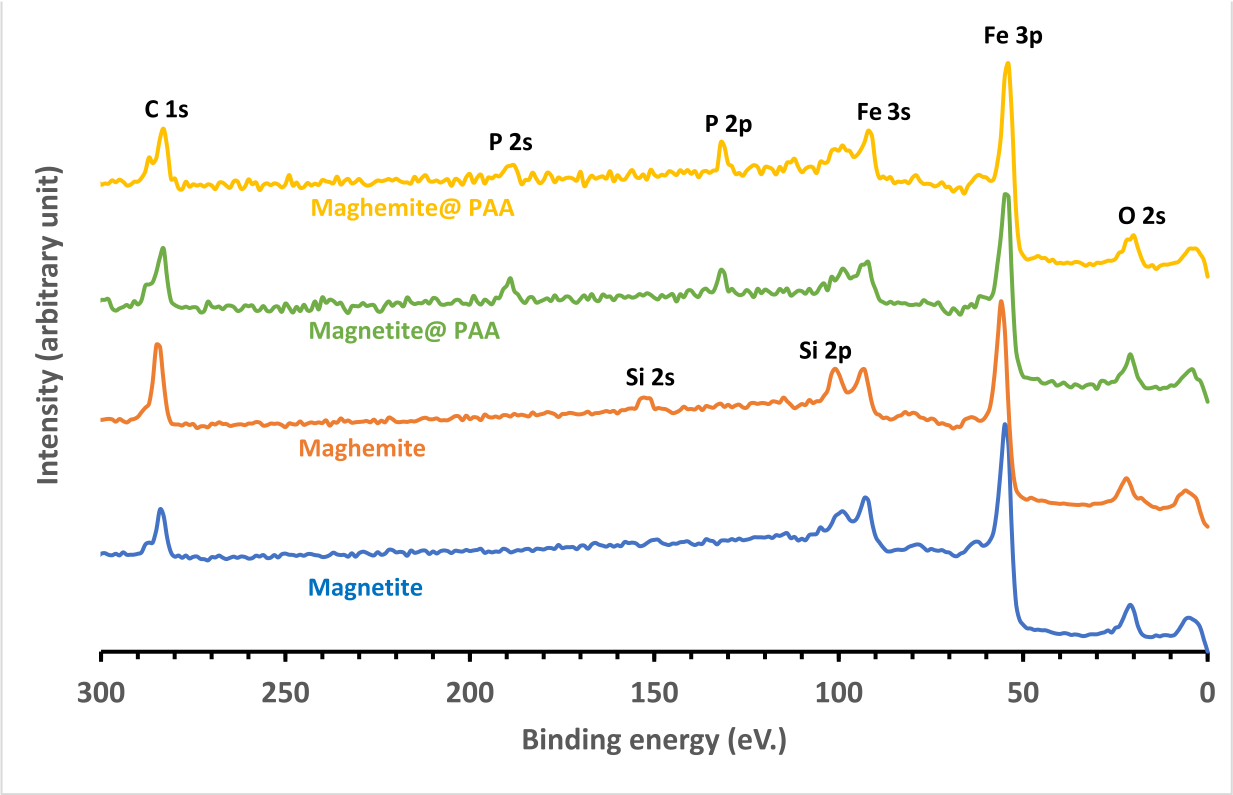

Fe 3p
C 1s
Fe 3s
P 2p
P 2s
O 2s
Maghemite@ PAA
Magnetite@ PAA
Si 2p
Si 2s
Maghemite
Magnetite
Intensity (arbitrary unit)
300
250
200
150
100
50
0
Binding energy (eV.)

(b)

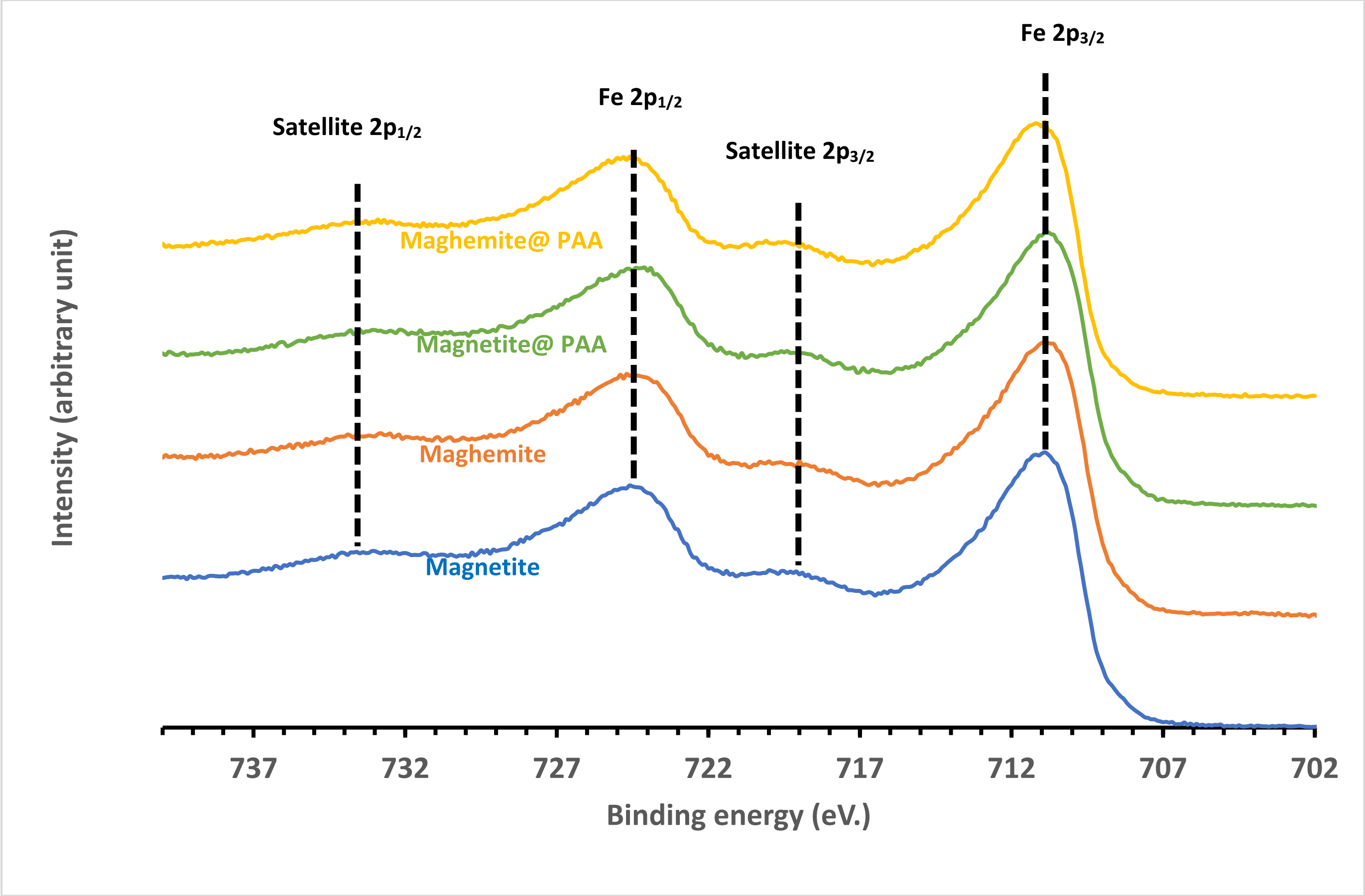

Fe 2p3/2
Fe 2p1/2
Satellite 2p1/2
Satellite 2p3/2
Maghemite@ PAA
Magnetite@ PAA
Maghemite
Magnetite
Intensity (arbitrary unit)
737
732
727
722
717
712
707
702
Binding energy (eV.)

(c)

	Fe $2p_{3/2}$	$2p_{3/2}$ satellite	Δ ($2p_{3/2}$ satellite -Fe $2p_{3/2}$)
Magnetite	711.0 ± 0.1	719.4 ± 0.1	8.4 ± 0.2
Maghemite	711+1 ± 0.1	719.4 ± 0.1	8.3 ± 0.2
Magnetite@PAA	711.0 ± 0.1	719.4 ± 0.1	8.4 ± 0.2
Maghemite@PAA	710.8 ± 0.1	719.4 ± 0.1	8.6 ± 0.2

(**d**)

Figure S2: Raman spectra of the AuNPs suspensions functionalized with MHA and PEG

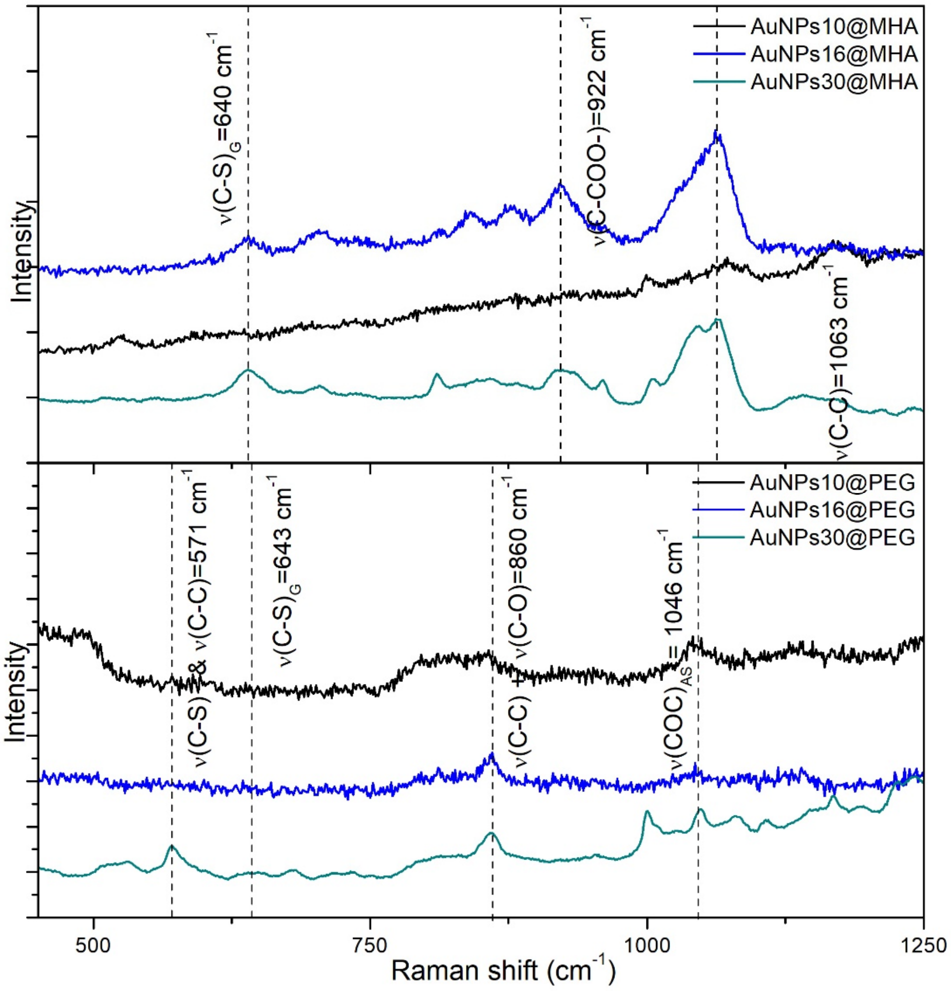

It was noticed during the analysis that the SERS effect is much more important with 30 nm AuNPs, which explains the absence of signal noise on the involved curves compared to the others.

Figure S3: XPS spectra of (a) AuNPs-30 and AuNPs30@PEG and (b) high-resolution spectra of C 1s and Au 4f of these samples.

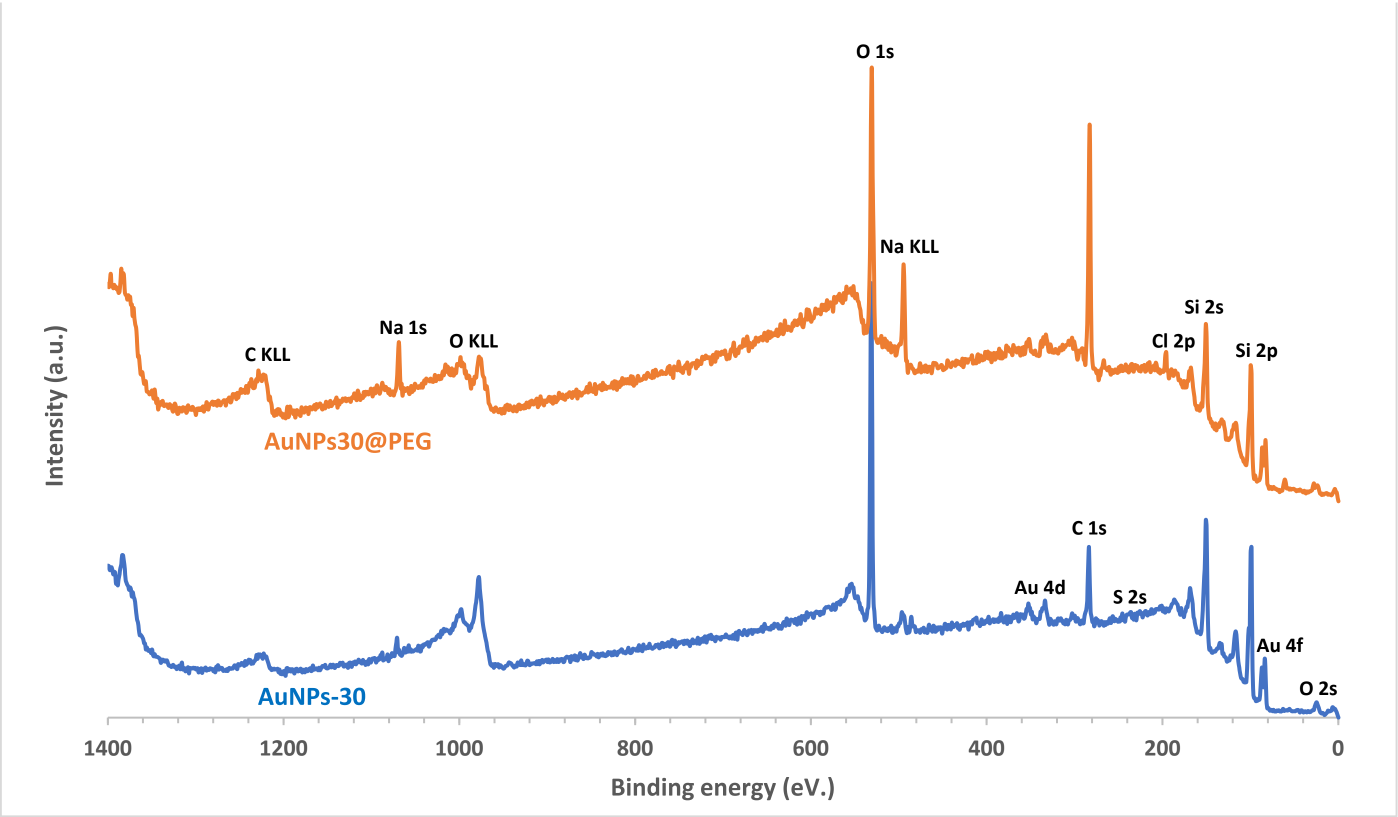

(**a**)

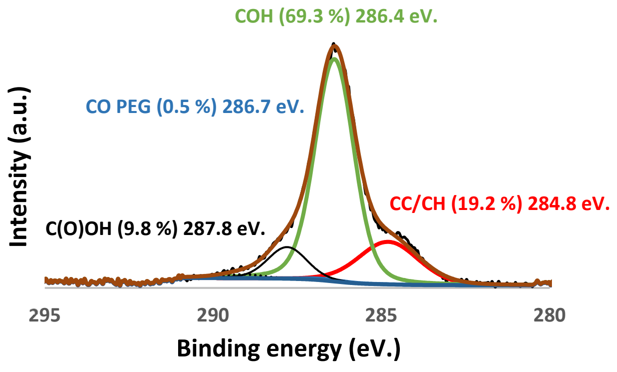

C 1s of AuNPs-30

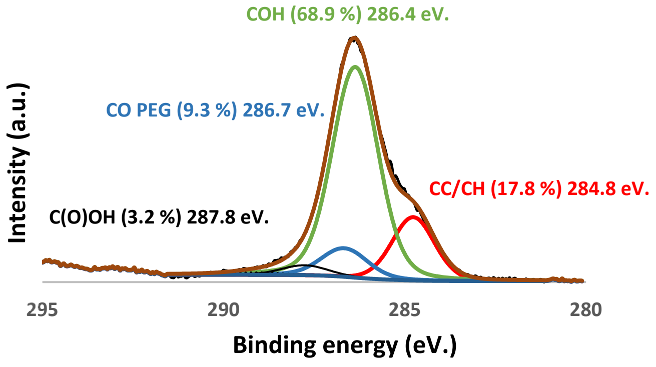

C 1s of AuNPs30@PEG

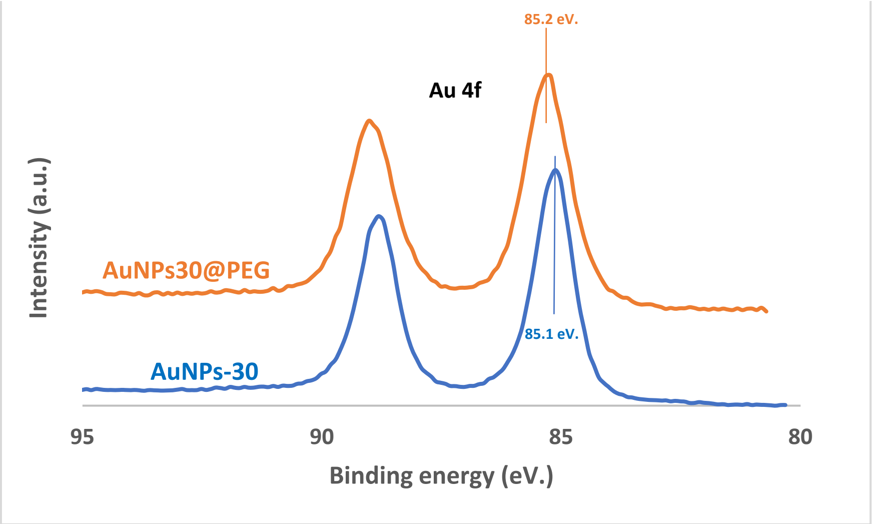

Au 4f high resolution spectra of AuNPs-30 and AuNPs30@PEG

(**b**)

Table S1: Precision for the calculation of PAA densities on functionalized SPIONs from ICP-OES

Sample	**C_{Fe} (mg/mL)**	**C_{SPIONs} (mg/mL)**	**C_{SPIONs} (NPs/mL)**	**C_P (mg/mL)**	**C_{PAA} (molec/mL)**	**PAA/SPIONs (nb)**
Fe_3O_4@PAA	0.57	0.78	1.1 x 10^{13}	0.012	2.2 x 10^{17}	7
γ- Fe_2O_3@PAA	0.63	0.91	8.6 x 10^{13}	0.013	2.6 x 10^{17}	4

Fe and P concentrations are calculated from a mean value of ICP-OES measurements at 3 different wavelengths. Conversion in iron oxide content is based on theoretical chemical formulae, and conversion to number of NPs is based on NPs volume calculated with DLS number size.

Figure S4: AFM image of the albumin layer spotted by Au10@PEG NPs and zoom-in on a 2D pack of auto-assembled NPs, with height measurement

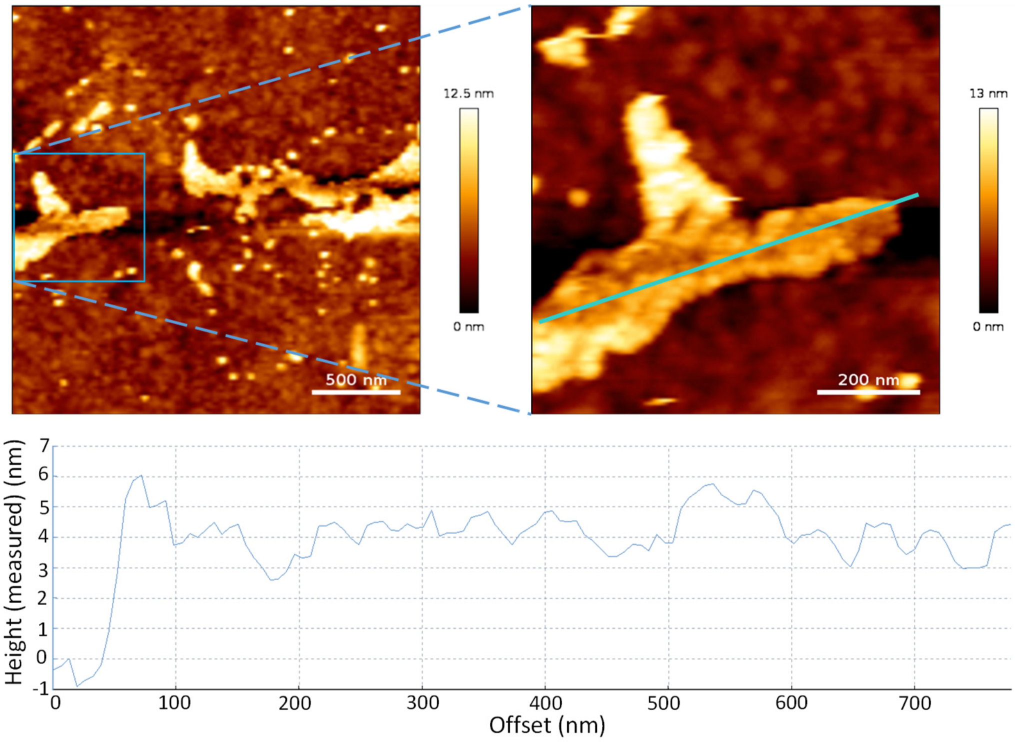

Along a 700 nm line, the measured heights on the auto-assembled pack of particles have differences of ±2 nm. This indicates a close packing of NPs, assembled in a 2D monolayer, so only the AuNPs@PEG grafted to the albumin remains on the surface.

Figure S5: (a) Average heights measured on the visualized objects of AFM images of the spotted 30 nm NPs (Figure 10) and (b) density of NPs observed on the surface

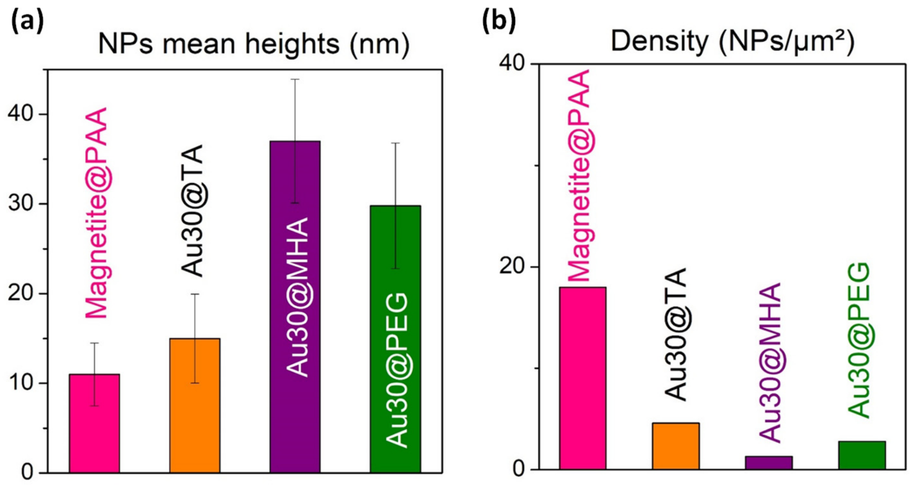

Due to the initial condition of spotting in identical NPs mass concentration, resulting number concentration of NPs is lower for bigger sized ones. It is thus difficult to get representative heights and densities with such low object concentration, increasing the analysis time. Those observations could then be more relevant with identical concentration in term of number of NPs.
